# Whole Genome Analysis of Three Multi-Drug Resistant *Listeria innocua* and Genomic Insights Into Their Relatedness With Resistant *Listeria monocytogenes*

**DOI:** 10.3389/fmicb.2021.694361

**Published:** 2021-07-23

**Authors:** Menghan Li, Shaofei Yan, Séamus Fanning, Fengqin Li, Jin Xu

**Affiliations:** ^1^NHC Key Laboratory of Food Safety Risk Assessment, China National Center for Food Safety Risk Assessment, Beijing, China; ^2^UCD-Centre for Food Safety, School of Public Health, Physiotherapy and Sports Science, University College Dublin, Dublin, Ireland

**Keywords:** *Listeria innocua*, multi-drug resistance, whole genome sequencing, listeria pathogenicity island 4, genome environment analysis

## Abstract

*Listeria innocua* are Gram-positive rod-shaped bacteria, which are not generally infectious as opposed to *Listeria monocytogenes*. However, the comparatively high genomic similarity between both along with on occasion, their coexistence in similar ecological niches may present the opportunity for resistance or virulence gene transfer. In this study, three multi-drug resistant *L. innocua* originally cultured from food were put forward for long-read genome sequencing. Chromosome and plasmid genomes were assembled and annotated. Analysis demonstrated that the resistant phenotypes correlated well with genotypes. Three plasmids pLI42, pLI203, and pLI47-1 were identified which harbor resistance islands. Sequence alignments suggested that plasmids pLI42 and pLI203 were highly similar to a previously sequenced *L. monocytogenes* plasmid pLR1. Similarly, another three types of resistance gene islands were observed on chromosome, including *tet*(M) gene islands (transposon Tn*916* orthologs), *dfrG* gene islands and *optrA-erm*(A) gene islands. All three *L. innocua* isolates possessed listeria pathogenicity island-4 (LIPI-4) which is linked to cases of mengitis. Further genome environment and phylogenic analysis of regions flanking LIPI-4 of *L. innocua* and *L. monocytogenes* showed that these may have common origins and with the potential to transmit from the former. Our findings raise the possible need to include both *L. monocytogenes* and *L. innocua* in food surveillance programs so as to further understand of the origins of antimicrobial resistance and virulence markers of public health importance in *L. monocytogenes.*

## Introduction

*Listeria* species are Gram-positive and facultative anaerobic bacteria that exist in soil, water and the animal gut. Members of this genus are found to contaminate certain types of foods and the associated food processing environment thereby representing a risk for public health (Finlay, 2001). Nineteen species of *Listeria* have been reported (Orsi and Wiedmann, 2016). Among them, *L. monocytogenes* is considered as the only one that can cause listeriosis both in humans and animals (Vivant et al., 2013). Previously, *Listeria innocua* was generally considered as a non-virulent species with a closer evolutionary relationship than other members of the genus to *L. monocytogenes* (Buchrieser et al., 2003).

To better describe the genomic evolution and potential for horizontal gene transfer (HGT) between *L. monocytogenes* and *L. innocua*, the findings of comparative genomics analyses were reported in previous studies (Buchrieser et al., 2003; Hain et al., 2006). These data highlighted the close genetic relationship existing between *L. monocytogenes* and *L. innocua*. Phylogenetic studies using amplicons or *Listeria* house-keeping genes provided evidence that *L. innocua* and *L. monocytogenes* are indeed related genetically (Glaser et al., 2001b; Orsi and Wiedmann, 2016). The orthologous genes identified between both species are highly conserved. Further, data indicated that *L. innocua* may have evolved through gene elimination and acquisition from the same pathogenic ancestor of *L. monocytogenes* (Chen et al., 2009).

Although few in number, early studies described plasmid-mediated AMR and their transmission in *L. innocua* (Bertsch et al., 2014; Gomez et al., 2014; Escolar et al., 2017). Based on recent sequencing data all *L. monocytogenes* possessed listeria pathogenicity island-1 (LIPI-1) and *inlAB* (Reddy and Lawrence, 2014). Hypervirulent isolates of *L. monocytogenes* harbor LIPI-3 and LIPI-4 (Maury et al., 2016), in which LIPI-3 encodes listeriolysin S, a second hemolysin that enhances the survival of *L. monocytogenes* in polymorphonuclear neutrophils (PMN) while LIPI-4 encodes a cellobiose-family phosphotransferase system (PTS) (Cotter et al., 2008), that enhances invasion of the central nervous system (CNS) along with maternal-neonatal infection (MN). Most *L. innocua* isolates lack LIPI-1 and several important virulence genes including *inlA and inlB*, while other data reported on atypical *L. innocua* that harbored LIPI-1 or LIPI-3 (Volokhov et al., 2007; Clayton et al., 2014; Moura et al., 2019). Unlike other LIPIs, LIPI-4 orthologous has been reported to be found in many *L. innocua* isolates (Moura et al., 2019). Furthermore, few examples of HGT involving resistance and virulence genes between these two species have been reported (Bertsch et al., 2014).

In this study, we describe three MDR *L. innocua* LI42, LI47, and LI203, isolated from food samples in China. In order to extend our understanding of the genetic relationships and antibiotic resistance and virulence, all three were sequenced and compared with the closely related *L. innocua* and *L. monocytogenes* reference genomes, including two resistant *L. monocytogenes* in our previous study (Yan et al., 2019).

## Materials and Methods

### Bacterial Isolates

Three *L. innocua isolates* were isolated from food in China from year 2015 to 2016. The source information was listed in Supplementary Table 1. All isolates were confirmed by API listeria (Suarez et al., 2001).

### Antibiotics Susceptibility Testing (AST)

All isolates were tested for antimicrobial susceptibility using broth microdilution against a panel of nine antimicrobial compounds commonly used in veterinary and human therapy and these data were interpreted according to the guidelines of the Clinical and Laboratory Standards Institute (CLSI) M45 (3rd edition) (Clsi Institute, 2015), where appropriate. Drugs tested included ampicillin (AMP), chloramphenicol (CHL), ciprofloxacin (CIP), erythromycin (ERY), gentamicin (GEN), meropenem (MEM), trimethoprim-sulfamethoxazole (SXT), tetracycline (TET), and vancomycin (VAN). All antibiotics were purchased from Sigma-Aldrich, Germany.

### DNA Purification and Sequencing

Each isolate was grown in brain heart infusion (BHI) broth (Beijing Land Bridge) at 37°C and genomic DNA (gDNA) was purified using Omega EZNA^®^ Bacterial DNA Kit (Omega Bio-tek, Norcross, GA, United States). The bacterial genomes were sequenced by Tianjin Biochip Corporation, using a PacBio RS II platform (Pacific Biosciences, Menlo Park, CA, United States). The sequencing depth is 1000X. *De novo* assembly was performed by SMRT Link (V6.0.0.47841).

### Annotation of Genomes and AMR Genes

The chromosomes and plasmids of three *L. innocua* were annotated with the prokaryotic genome annotation tool Prokka (v1.12). Antibiotic resistance genes were extracted from these genome sequences using the ABRicate^1^ software package, where a combination of three reference databases CARD (Jia et al., 2017), ResFinder (Zankari et al., 2012), and NCBI AMRFinderPlus (Feldgarden et al., 2019) were used. Gene names were unified to the NCBI AMRFinderPlus references. All resistance genes were screened using the BLASTN algorithm with minimum nucleotide identity and alignment length coverage of 80%.

### Assessment of Virulence Factors

The presence and integrity of virulence factors was assessed using *L. monocytogenes* EGD-e (NC_003210) as the reference genome for Internalin A (*InlA*), Internalin B (*InlB*), listeria pathogenicity island 1 (LIPI-1) (Glaser et al., 2001b; Toledo-Arana et al., 2009). *L. monocytogenes* F2365 (NC_002973) was used as reference genome for LIPI-3 with the protein sequences (LMOf2365_1113 to LMOf2365_1119) (Nelson et al., 2004). *L. monocytogenes* LM9005581 (CYWW00000000) was used as reference for LIPI-4 with the protein sequences (LM9005581_70009 to LM9005581_70014). Analysis was performed using the BLASTN algorithm with a minimum identity of 80%, coverage of 80%.

### Genomic Comparison

Sequence comparison was executed between chromosomes or plasmids on average nucleotide identity (ANI) based on BLASTN alignment, using pyani (v0.2.7). ANIb values of each pair of samples was calculated and classified into two groups *via* species. A one-tailed student *t*-test was performed. A circular genome comparison graph was performed with BRIG (v0.95). Sequence comparisons were done using BLASTN and visualized using EasyFig (v2.2.3).

### Core Genome Alignment and Phylogenetic Tree

Core genomes of all assemblies were calculated using Roary (v3.11.0). The core genomes were aligned with MAFFT (v7.313). Maximum Likelihood phylogenetic tree of the aligned genomes was performed using FastTree (v2.1.10). The phylogenetic tree was illustrated by adjusting the mid-point as root.

### Sequence Data Accession Numbers

Accession numbers for complete genome sequences are SAMN18079989 (LI42), SAMN18080006 (LI47), and SAMN18080009 (LI203).

### Conjugation Experiments

Conjugation experiments were performed using *L. innocua* LI42, LI47, and LI203 as the donors, *L. monocytogenes* ATCC 19115, *L. monocytogenes* ST9 isolate and *E. coli* J53 (NaN_3_ resistant) as recipients. For the selection of the transconjugants between *L. innocua* and *L. monocytogenes*, blood agar plate was supplemented with 4 mg/L tetracycline, and the colonies were separated by hemolysis test. For the selection of the transconjugants between *L. innocua* and *E. coli* J53, MacConkey Agar (MAC) plate was supplemented with 4 mg/L tetracycline and 100 mg/L NaN_3_. The Colonies grew on these selective plates were further confirmed by PCR amplification of *hly* and *tet*(S) genes for *listeria spp*., and *tet*(S) for *E. coli* J53.

## Results

### Pheno- and Genotypic Characterization of Three *L. innocua* Isolates

**FIGURE 1:**
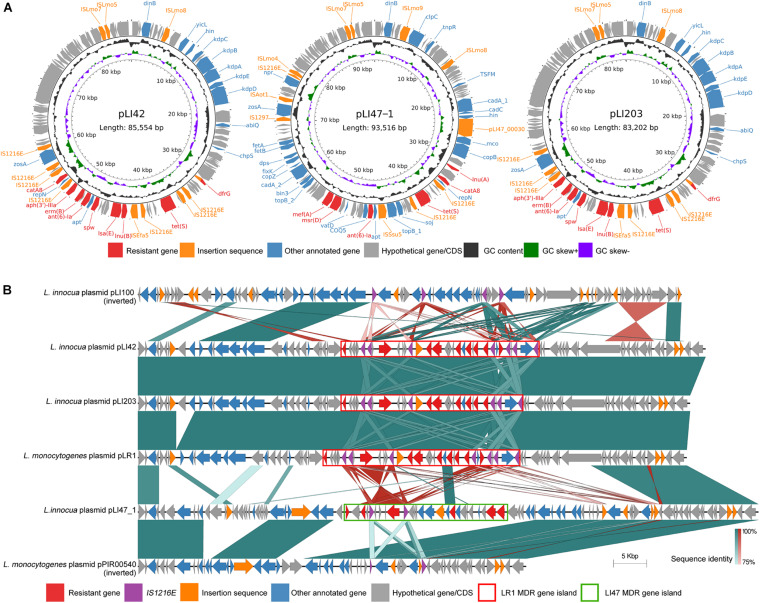
Circular schematic map and linear sequence comparison of three plasmids carrying antibiotic resistance genes. **(A)** The sequence structure of three sequenced *L. innocua* plasmid pLI42, pLI47-1, and PLI203. CDSs were shown as arrows. Antibiotic resistance genes were highlighted as red. Insertion sequences were highlighted by orange. Other annotated genes were denoted by blue. The inner two rings represented GC Skew[(G – C)/(G + C), G and C represents guanine content cytosine content] and GC content. **(B)** Comparison of three *L. innocua* plasmid pLI47-1, pLI203, and PLI42 sequences with a closely related reference *L. innocua* plasmid pLI100 and two *L. monocytogenes* reference plasmid pLR1 and pPIR00540. CDSs were shown as arrows and colored based on gene functions. Copies of insertion sequence IS*1216E* was specially highlighted as purple. Green or red shades between each two sequences represented direct or inverted nucleotide identity between covered regions with at least 75% identity. Reference plasmids pLI100 and pPIR00540 sequences were reversed to be aligned with other sequences. *L. monocytogenes* LR1 MDR gene island and the orthologs were marked in red frames. *L. innocua* LI47 MDR gene island was marked in green frame.

*Listeria innocua* LI42, LI47, and LI203 were found to express the same MDR phenotype resistant to chloramphenicol, erythromycin, tetracycline and trimethoprim-sulfamethoxazole as shown in Figure 1, albeit somewhat different minimum inhibitory concentration (MIC) values. AMR genotypes corresponded well with the phenotypes described by AST analysis, where the three MDR isolates harbored resistance genes, respectively, for aminoglycoside, macrolide-lincosamide-streptogramin B (MLS_b_), phenicol, tetracycline, and sulfamethoxazole resistance. Complete LIPI-4 orthologs were found in all three *L. innocua* isolates.

### Genome Wide Characterization of Three *Listeria innocua* Genomes

As shown in Table 1, long-read sequencing of *L. innocua* LI42, LI47, LI203 facilitated the construction of the complete genome sequence in each case including their chromosomes and plasmids. *Listeria innocua* LI42 and LI203 genomes each contain one chromosome and one plasmid (pLI42 or pLI203), while *L. innocua* LI47 contained a chromosome and two plasmids (pLI47-1 and pLI47-2).

**TABLE 1:** Summary for complete genome sequencing and antimicrobial resistance pheno- and genotypes of three *L. innocua* isolates.

**Isolate**	**Sequence type**	**Total length (bp)**	**GC content (%)**	**Antibiotic resistance genes**	**MLST**	**Antibiotic susceptibility (MIC, μg/mL)**								
						**AMP**	**CHL**	**CIP**	**ERY**	**GEN**	**MEM**	**TET**	**SXT**	**VAN**
LI42	Chromosome	2,930,429	37.45	*tet*(M), *optrA, fexA*	474	S (0.25)	R (>128)	S (1)	R (32)	S (0.25)	S (0.25)	R (32)	R (4/76)	S (0.5)
	Plasmid (pLI42)	85,554	34.64	*dfrG, tet*(S), *lnu*(B), *lsa*(E), *ant*(6)*-Ia, erm*(B), *aph(3′)-IIIa, catA8*										
LI47	Chromosome	2,927,254	37.46	*erm*(A), *dfrG,optrA, fexA*	602	S (0.25)	R (>128)	S (1)	R (32)	S (1)	S (0.25)	R (16)	R (2/38)	S (0.5)
	Plasmid (pLI47-1)	93,516	35.99	*lnu*(A), *catA8, tet*(S), *ant*(6)*-Ia, msr*(D), *mef*(A)										
	Plasmid (pLI47-2)	52,798	31.63	*–*										
LI203	Chromosome	2,940,225	37.47	*tet*(M), *optrA, fexA*	474	S (0.5)	R (>128)	S (1)	R (16)	S (0.25)	S (0.1)	R (64)	R (2/38)	S (1)
	Plasmid (pLI203)	83,202	34.77	*dfrG, tet*(S), *lnu*(B), *lsa*(E),*ant(6)-Ia, erm*(B), *aph(3′)-IIIa, catA8*										

*The table shows basic information summary of three *L. innocua* samples and their genome sequences.*

*The abbreviations were ampicillin (AMP), chloramphenicol (CHL), ciprofloxacin (CIP), erythromycin (ERY), gentamicin (GEN), meropenem (MEM), trimethoprim-sulfamethoxazole (SXT), tetracycline (TET), and vancomycin (VAN).*

*Antibiotic susceptibilities are described as S (susceptible)/R (resistant) with the original minimum inhibitory concentration (MIC) value.*

*MIC breakpoints of AMP, SXT, and MEM were in accordance with CLSI M45 (3rd edition), all other compounds were interpreted using *Bacillus* spp. as the reference from the same document.*

### Comparative Genomic Analysis of Three *Listeria innocua* Plasmids

Annotation of resistance genes showed that plasmid pLI42, pLI47-1, and pLI203 carried multiple antibiotic resistance genes including *ant*(6)*-Ia, aph(3′)-IIIa, catA8, dfrG, erm*(B), *lnu*(A), *lnu*(B), *lsa*(E), *msr*(D), *mef*(A), *spw* and *tet*(S), associating resistances of amikacin, aminoglycoside, chloramphenicol, kanamycin, lincosamide, macrolide, tetracycline, trimethoprim, streptogramin, and streptomycin. Multiple copies of insertion sequences were also noted (Figure 1A).

Our previous study reporting on *L. monocytogenes* from foods in China described a multidrug resistant (MDR) gene island *dfrG-tet*(S)-*lnu*(B)*-lsa*(E)*-spw-ant*(6)*_Ia-erm*(B)*-aphA-catA8* from *L. monocytogenes* LR1 (SAMN10434273) (Yan et al., 2019). The recent long-read re-sequencing of this strain confirmed that this gene island is located on a plasmid, denoted as plasmid pLR1. Average nucleotide identities between each pair of plasmids pLI42, pLI203 and *L. monocytogenes* MDR plasmid pLR1 sequences showed >95.0% coverages and >99.9% identities between each two sequences, demonstrating that plasmids pLI42 and pLI203 are close orthologs of plasmid pLR1.

Linear sequence comparison was performed involving plasmids pLI42, pLI47-1, and pLI203 along with three plasmids pLI100 (*L. innocua*) from *L. innocua* Clip11262 (Glaser et al., 2001a), pLR1 (*L. monocytogenes*), and pPIR00540 (*L. monocytogenes*) as the closest common hits of online BLAST result of the three plasmids against NCBI nt/nr database (Portmann et al., 2018), which clearly displayed the ortholog regions (Figure 1B). Although plasmid pLI203 lacked the chloramphenicol resistance gene *catA8*, plasmids pLI42, pLI203, and pLR1 shared a similar overall genetic backbone covering the previously described LR1 MDR gene island. *Listeria innocua* LI47 MDR gene island mapped to plasmid pLI47-1 demonstrated a unique resistance gene arrangement *lnuA-catA8-tet*(S)*-ant*(6)*Ia-msr*(D)*-mef*(A). The conserved flanking region of *L. innocua* LI47 MDR gene island on plasmid pLI47-1 was identical to plasmid pPIR00540.

Insertion sequence (IS) elements IS*3*, IS*6*, IS*21*, IS*1595*, IS*1380*, IS*Lre2*, and Tn*3* were also annotated, along with the resistance gene *dfrG*, which is also known as transposes IS*Ssu9* (Holden et al., 2009). Multiple copies of IS*1216E* were noted on these plasmids. Each of plasmids pLI42, pLI203, and pLR1 had six direct repeats of IS*1216E*. Plasmids pLI47-1 and pPIR00540 contain two direct repeats, while three direct repeats and a single inverted repeat are found in plasmid pLI100.

### Comparative Genomic Analysis of Resistance Gene Islands Located on Chromosomes

*Listeria innocua* LI42, LI47, and LI203 chromosomes were discovered harboring three different types of resistance gene island including *tet*(M) gene island (∼10,925 bp, found on the LI42 and LI203 chromosomes, associated with tetracycline resistance), *dfrG* gene island (∼3,310 bp, mapped on the LI47 chromosome, and associated with trimethoprim resistance) and *optrA-erm*(A) gene island (∼18,861 bp, integrally found on the LI47 chromosome, partially found on LI42 and LI203 chromosome, associated with macrolide, florfenicol and oxazolidinone resistance).

**FIGURE 2:**
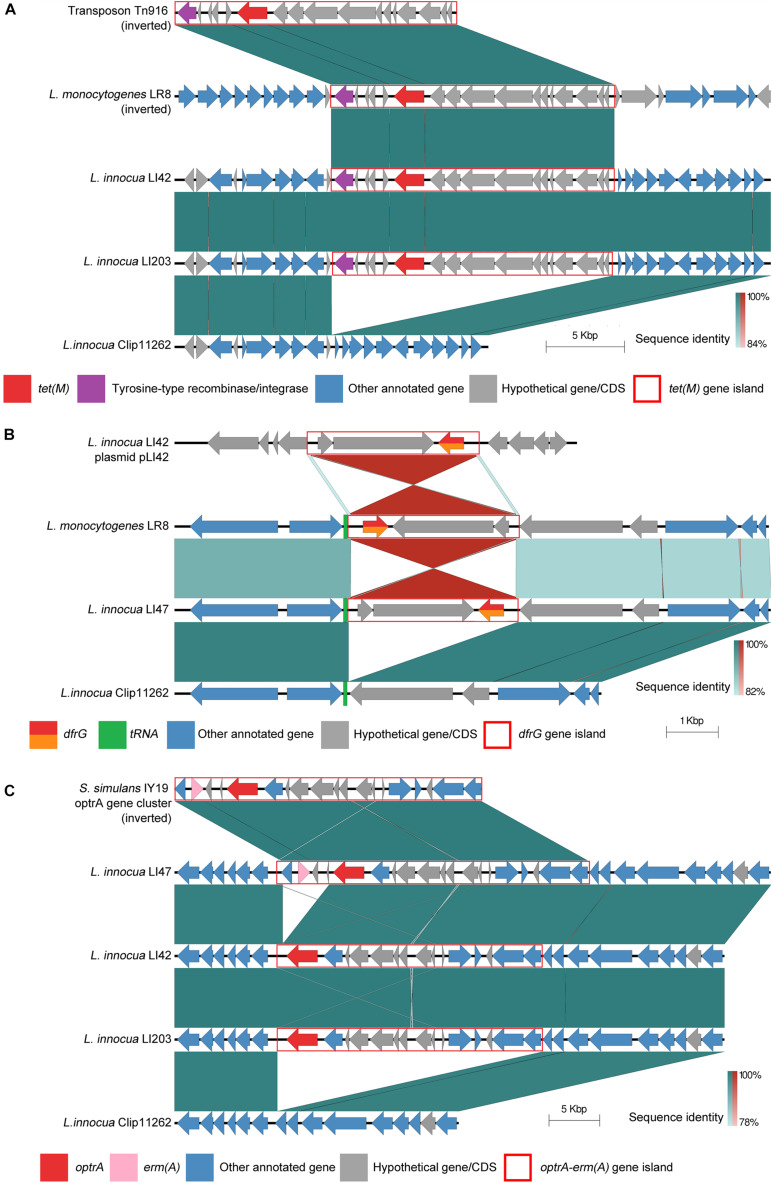
Linear sequence comparison of resistance genes and its flanking region on chromosome. The *tet*(M) gene islands **(A)**, *dfrG* gene islands **(B)**, and *optrA-erm*(A) gene islands **(C)** and their flanking regions of LI42, LI47 or LI203 chromosomes was compared with their most closely related orthologs from nr/nt database and the susceptible *L. innocua* reference genome Clip11262. **(A)** Comparison of the *tet*(M) gene islands on LI42 and LI203 chromosomes with *L. monocytogenes* LR8 and transposon TN916. **(B)** Comparison of *dfrG* gene island in LI47 chromosome with plasmid pLI42 and *L. monocytogenes* LR8 chromosome. **(C)** Comparison of *optrA-erm*(A) gene islands in LI47, LI42, and LI203 chromosomes with *S. simulans* IY19 optrA gene cluster. Resistance genes and genes related to insertion or recombination were highlighted. Resistance gene islands were marked in red frames. Green or red shades between each two sequences represented direct or inverted nucleotide identity between covered regions.

Nucleotide comparison confirmed that the *tet*(M) and *dfrG* gene-containing islands are highly identical (>99%) with their orthologs in *L. monocytogenes* LR8 (SAMN10434278), a feature reported in our earlier study (Yan et al., 2019). Specifically, *tet*(M) is an identical ortholog of transposon Tn*916*, which is found among several different bacteria (Kathariou et al., 1987; Bertsch et al., 2013), while the *dfrG* gene is known to exist as an independent antibiotic resistance gene and also associated with an insertion sequence element IS*1595*. The insert locations for the *tet*(M) gene-containing island in *L. innocua* LI42 and LI203 chromosomes differ from that of *L. monocytogenes* LR8 (as shown in Figure 2A). Meanwhile, the *dfrG* gene-containing island also mapped to plasmids pLI42 and pLR1, suggesting horizontal movement between both plasmids and chromosomes. The latter contains a pair of short direct repeats (∼63 bp) flanking both ends, and module inserts reversibly at the corresponding location on the chromosomes of *L. innocua* LR8 and LI42 (Figure 2B).

The *L. innocua* LI42 chromosome harbors the complete *optrA-erm*(A) gene island. It is identical to *Staphylococcus simulans* IY19 *optrA* gene cluster (MF805730), which was reported in an earlier study (Sun et al., 2018). In *L. innocua* LI47 and LI203 a partial *optrA-erm*(A) gene island only was noted, and which is devoid of *erm*(A) gene. The insertion locations of this gene island were consistent among *L. innocua* LI42, LI47 and LI203. Flanking regions of all three resistance gene islands were identical in comparison with the susceptible *L. innocua* reference genome Clip11262.

### Genome Environment Analysis of LIPI-4

**FIGURE 3:**
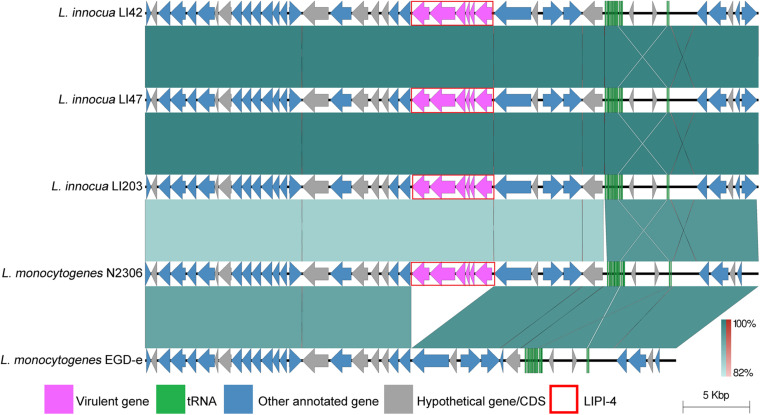
Linear sequence comparison of LIPI-4 and its flanking regions. Sequences of LIPI-4 and their flanking regions on LI42, LI47, and LI203 genomes were compared with their ortholog in *L. monocytogenes* reference genome N2306 and EGD-e. Virulence genes were highlighted in pink arrows and LIPI-4 regions were marked in red frame. Green or red shades between each two sequences represented direct or inverted nucleotide identity between covered regions.

Genomic comparisons were performed on LIPI-4 and its flanking region in *L. innocua* and *L. monocytogenes* in order to discover the degree to which it may be conserved between these species. The LIPI-4 ortholog was located at the corresponding position on the chromosomes of *L. innocua* LI42, LI47,and LI203 and the reference *L. monocytogenes* N2306. The sequence context of LIPI-4 was found to be consistent. The reference *L. monocytogenes* EGD-e was devoid of LIPI-4, while possessing an identical LIPI-4 flanking region (as shown in Figure 3).

A 10-kbp upstream and downstream region flanking LIPI-4 were assessed for a comparative analysis of their genomic features. These regions were found to be identical among the *L. innocua* strains, and in *L. monocytogenes* with/without LIPI-4. The LIPI-4 locus of both species shared an identical *lacE-celA-lacF-chbG-manR-pagL* gene arrangement. Average sequence identity of each gene within/between species is shown in Supplementary Table 2. *L. monocytogenes* lacking LIPI-4 demonstrated highly similar orthologous regions to the LIPI-4 adjacent regions, especially for the lineage-I strains. The sequence identity between the non-LIPI-4 orthologs and the LIPI-4 containing *L. monocytogenes* lineage-I was approximately 100%.

## Discussion

### Genomic Features of Antibiotic Resistance

In this study, three *L. innocua* each expressing an MDR phenotype were studied. When sequenced, their corresponding antibiotic resistant-encoding genes were found to map either to the bacterial chromosome or to plasmids contained therein. In the case of these plasmids that were identified, an ortholog of *L. monocytogenes* MDR-expressing plasmid pLR1 were detected in *L. innocua* LI42 and LI203. The same plasmid in a *L. monocytogenes* isolate was also recovered in China, and denoted as plasmid pNH1 (Yan et al., 2020). A novel MDR expressing plasmid pLI47-1 is also described in this study. Sequence comparisons highlighted the mosaic nature of MDR gene islands in both of these plasmids wherein their resistance genes appeared to arise from multiple origins including *Staphylococcus aureus*, *Enterococcus faecium*, and *Lactococcus lactis*, with IS1*216E* likely to play a decisive role in the recombination steps contributing to their formation. Similar observations have been reported earlier by others (Kang et al., 2019; Moroi et al., 2019; Iimura et al., 2020).

The >99% sequence identities between plasmid pLI42 and pLI203 in *L. innocua* and pLR1 in *L. monocytogenes* showed that these plasmids were highly homologous. Meanwhile, the isolates harboring these plasmids were isolated from different years, for *L. monocytogenes* isolate LR1 harboring pLR1 was discovered in 2012, while the *L. innocua* isolates harboring pLI42 and pLI203 were discovered in 2015 and 2016. These evidences may indicate the potential mobilizing nature of these plasmids between *Listeria* species. However, our conjugation experiment showed that pLI42, pLI47-1 and pLI203 in *L. innocua* were non-conjugative to *L. monocytogenes* or *E. coli* (data not shown). A previous study also reported that the homologous plasmid pNH1 was non-conjugative between *L. monocytogenes* strains (Yan et al., 2020). Thus, the specific mobilizing mechanism of the plasmids still needs to be revealed.

On chromosomes, three different types of antibiotic resistance gene-containing islands were discovered in *L. innocua* in this study. The *tet*(M) gene-containing islands were found to be orthologs of transposon Tn*916*. The differences in the sequence context identified in *L. monocytogenes* LR8 and the two *L. innocua* isolates suggested its ability to transfer and recombine at different positions on bacterial chromosome. The short direct repeat sequences flanking *dfrG* gene-containing islands may facilitate insertion into the genome, a feature noted on both chromosomes as well as plasmids. The flanking regions of *dfrG* islands in *L. innocua* LR8 and LI47 are identical, while the insertional orientation of the module is opposite, reflecting the flexibility of this step. There were no orthologs of *optrA-erm*(A) gene-containing islands found in any *L. monocytogenes* genomes analyzed to date. However, its identification in *S. simulans* (Sun et al., 2018), a non-closely related species of *L. innocua*, as well as their significant identity, suggested a potentially recent horizontal gene transfer, which possibly hints at its horizontal transmitting nature. Additionally, the GC content of *optrA-erm*(A) gene island is 35.1%. Comparing with *L. monocytogenes* (∼38.1%) and *L. innocua* (∼37.5%), it is closer to *S. simulans* (∼35.9%, *S. simulans* strain NCTC11046, NZ_LS483313). Thus *S. simulans* is more likely to be the original host of this gene island.

Discovery of *L. monocytogenes* MDR plasmids and resistance gene-containing islands on chromosome in *L. innocua* confirmed the fact that the latter can act as a gene sink, collecting AMR determinants from a range of sources. Antimicrobial resistant *L. innocua* have the potential to constitute a serious threat to public health through possible transferring of resistance genes to susceptible *L. monocytogenes*.

### Genomic and Evolutionary Features and of Virulence

*L. innocua* containing LIPIs are usually considered as “atypical”’ (Volokhov et al., 2007; Clayton et al., 2014; Moura et al., 2019). Nonetheless, LIPI-4 is recently reported widespread among this species (Moura et al., 2019). In this study, LIPI-4 was identified in all three *L. innocua* studied. The existence of the LIPI-4 orthologs in *L. innocua* suggested a possibility of gain or loss of virulence genes during evolution.

A LIPI-4 phylogenetic tree created using 10 *L. innocua* and 11 *L. monocytogenes* clearly clustered by species (Supplementary Figure 1). The tree was split precisely into two branches in accordance with their species. The *L. innocua* branch had longer internal evolutionary distances compared to the other, implying a longer evolutionary history. The core genome phylogenetic tree of *L. innocua* and *L. monocytogenes* indicated an explicit clustering by species. The *L. monocytogenes* branch was clustered by lineages, while *L. monocytogenes* LIPI-4 were interspersed in the *L. monocytogenes* lineage -I cluster (Supplementary Figure 2). All *L. monocytogenes* containing LIPI-4 were found in lineage -I, and in this case the branch containing *L. monocytogenes* lineages -II and -III collapsed. There was no explicit common ancestor for the *L. monocytogenes* containing LIPI-4.

To better discover the evolutionary pathway of LIPI-4, K_a_/K_s_ values were calculated for both *L. innocua* and *L. monocytogenes*, except wherein the gene had no mutation among all pairs (Supplementary Table 3; Wang et al., 2009, 2010). When the K_a_/K_s_ ratio was less than 1 for all *L. innocua* and monocytogenes LIPI-4 genes, this implied that LIPI-4 was under purifying selection for both species. All genes of LIPI-4 orthologous were in identical order and shared high similarity in gene sequence individually. All *L. monocytogenes* harboring LIPI-4 belonged to lineage -I. Both genomes and LIPI-4 containing regions of *L. monocytogenes* were less diverse comparing with those of *L. innocua*. Additionally, no LIPI-4-absent *L. innocua* strain had been reported. Combining the above observations, it may imply that LIPI-4 of both species may originate from same ancestor. The obviously slower differentiation rate of *L. monocytogenes* LIPI-4 than that of *L. innocua*, suggested *L. innocua* may acquire the LIPI-4 earlier than *L. monocytogenes*.

Since phylogeny of LIPI-4 and genome showed no branch crossing, together with the result of the sequence comparison where average identity of LIPI-4 was 4% lower than the overall genome average identity between the two species, it is less likely that horizontal gene transfer of LIPI-4 arose in from contemporary *L. innocua*. Moreover, as no other species besides *L. innocua* and *L. monocytogenes* have been found to harbor a LIPI-4 ortholog or found through online BLASTN toward NCBI nt/nr database. It could be assumed that LIPI-4 may have transferred from a progenitor of *L. innocua* to a later *L. monocytogenes* lineage -I. However, the origins and transferring path of LIPI-4 remains unknown.

## Conclusion

*L. innocua*, shared resistance and virulence genes with its infamous close relative *L. monocytogenes*, is not totally innocuous. As reports have highlighted, *L. innocua* and *L. monocytogenes* are commonly detected together in the same ecological niches (Franco et al., 1995; Wagner et al., 2007; Kim et al., 2017; Zhao et al., 2021). This implies the possibility of virulence/resistance gene transferring between these two species on the other side. Therefore, it would be better for public health that *L. innocua* need to be taken into consideration to refine the risk assessment of *L. monocytogenes* during future food surveillance and monitoring.

## Data Availability Statement

Accession numbers for complete genome sequences are SAMN18079989 (LI42), SAMN18080006 (LI47), and SAMN18080009 (LI203).

## Author Contributions

All authors listed have made a substantial, direct and intellectual contribution to the work, and approved it for publication.

## Conflict of Interest

The authors declare that the research was conducted in the absence of any commercial or financial relationships that could be construed as a potential conflict of interest.

## Publisher’s Note

All claims expressed in this article are solely those of the authors and do not necessarily represent those of their affiliated organizations, or those of the publisher, the editors and the reviewers. Any product that may be evaluated in this article, or claim that may be made by its manufacturer, is not guaranteed or endorsed by the publisher.

## Footnotes

1 https://github.com/tseemann/abricate

## Supplementary Material

Supplementary Figure 1 LIPI-4 phylogenetic tree of 4 *L. innocua* isolates with reference *L. innocua* and *L. monocytogenes*. Phylogenetic tree was built with FastTree and modified by FigTree using LIPI-4 sequences of 4 *L. innocua* and from NCBI nt/nr database. Root was set as midpoint.

Supplementary Figure 2 Genome phylogenetic tree of 4 *L. innocua* isolates with reference *L. innocua* and *L. monocytogenes*. Phylogenetic tree was built with FastTree (v2.1.10) and modified by FigTree using whole genome sequences of 4 *L. innocua* and completely assembled reference genomes of *L. monocytogenes* and *L. innocua* from NCBI assembly database. Root was set as midpoint. *L. innocua* strains were marked in blue. *L. monocytogenes* strains with LIPI-4 were marked in red, while the strains without LIPI-4 were marked in yellow.
