# Supplementary figures and images for: Whole Genome Analysis of Three Multi-Drug Resistant Listeria innocua and Genomic Insights Into Their Relatedness With Resistant Listeria monocytogenes
Source: Front Microbiol. 2021 Jul 23;12:694361. doi: 10.3389/fmicb.2021.694361 (PMC8343405; doi:10.3389/fmicb.2021.694361)

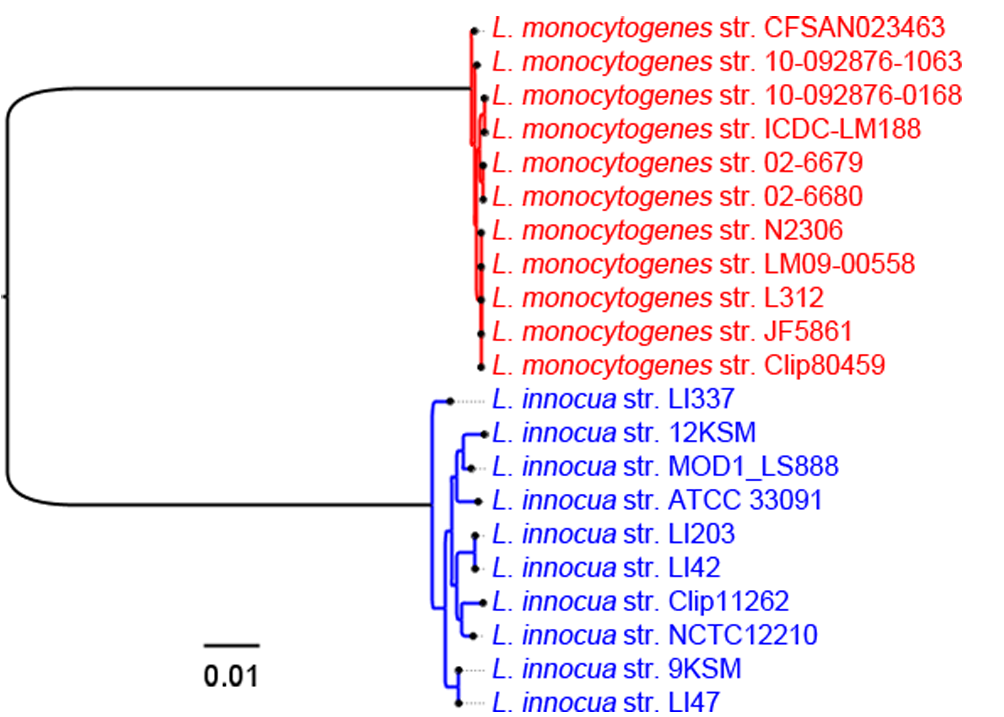

Supplement: Supplementary Figure 1 — LIPI-4 phylogenetic tree of 4 L. innocua isolates with reference L. innocua and L. monocytogenes. Phylogenetic tree was built with FastTree and modified by FigTree using LIPI-4 sequences of 4 L. innocua and from NCBI nt/nr database. Root was set as midpoint. [file Image_1.TIF]

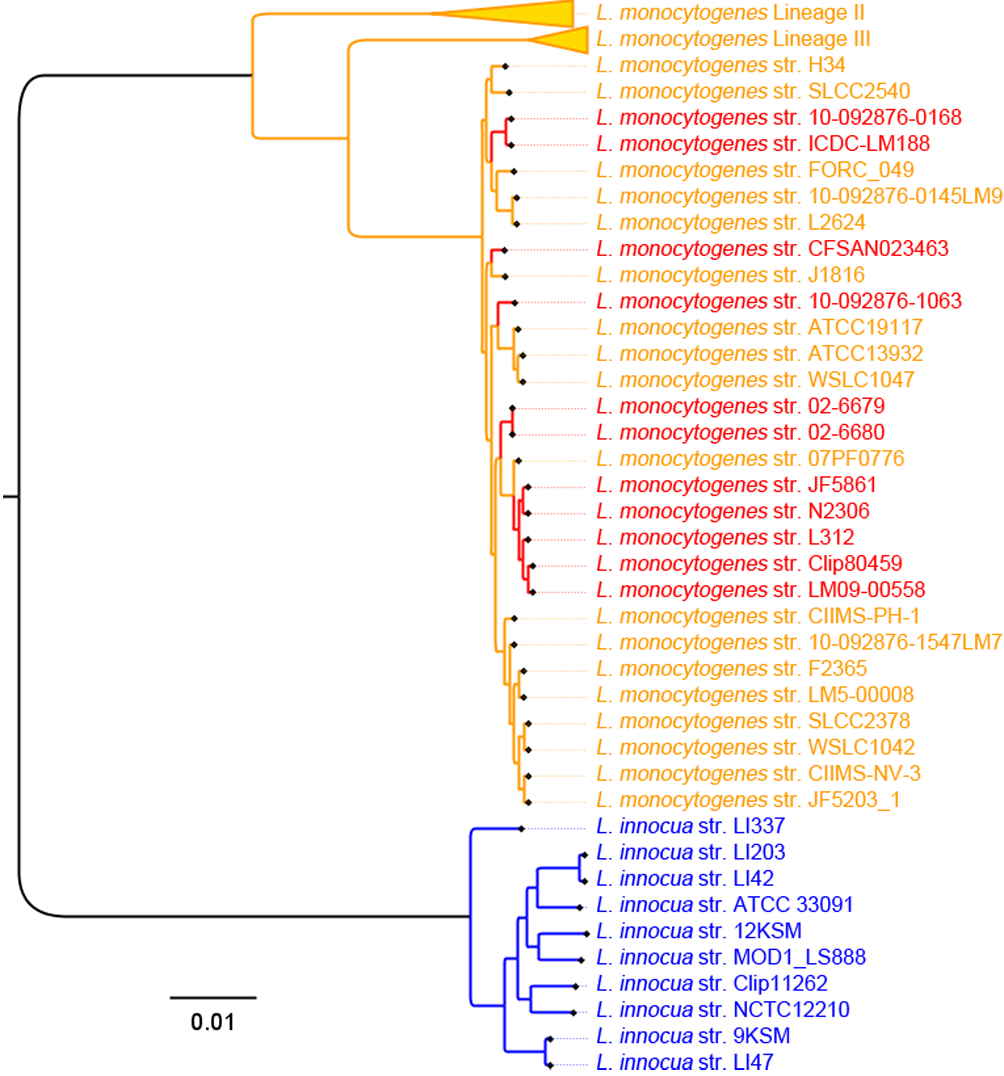

Supplement: Supplementary Figure 2 — Genome phylogenetic tree of 4 L. innocua isolates with reference L. innocua and L. monocytogenes. Phylogenetic tree was built with FastTree (v2.1.10) and modified by FigTree using whole genome sequences of 4 L. innocua and completely assembled reference genomes of L. monocytogenes and L. innocua from NCBI assembly database. Root was set as midpoint. L. innocua strains were marked in blue. L. monocytogenes strains with LIPI-4 were marked in red, while the strains without LIPI-4 were marked in yellow. [file Image_2.TIF]
