# Supplementary material for: Whole Genome Analysis of Three Multi-Drug Resistant Listeria innocua and Genomic Insights Into Their Relatedness With Resistant Listeria monocytogenes
Source: Front Microbiol. 2021 Jul 23;12:694361. doi: 10.3389/fmicb.2021.694361 (PMC8343405; doi:10.3389/fmicb.2021.694361)
Supplement: Supplementary file 3 [file Table_1.docx]

**Table S1** **Metadata of the 3 *L. innocua* isolates**

| Isolates | Year of isolation | Lineage | Serogroup | Food type | Food origin |
| --- | --- | --- | --- | --- | --- |
| LI42 | 2015 | *L. innocua* | L | raw meat | chicken |
| LI47 | 2015 | *L. innocua* | L | raw meat | pork |
| LI203 | 2016 | *L. innocua* | L | raw meat | fish |
