# Supplementary material for: Whole Genome Analysis of Three Multi-Drug Resistant Listeria innocua and Genomic Insights Into Their Relatedness With Resistant Listeria monocytogenes
Source: Front Microbiol. 2021 Jul 23;12:694361. doi: 10.3389/fmicb.2021.694361 (PMC8343405; doi:10.3389/fmicb.2021.694361)
Supplement: Supplementary file 4 [file Table_2.docx]

**Table S2 Open-sourced reference sequences used in this study.**

Thirteen *L. monocytogenes* and six *L. innocua* online assembly genome sequences were included for comparative genomics analysis along with the experimental *L. innocua* strains.

*L. monocytogenes* strain LM09-00558 (NCBI Biosample: SAMEA3540887; NCBI assembly accession: GCF_001565535.2) LIPI-4 sequence is used as the LIPI-4 reference in this study. A NCBI online BLASTN was executed against the nr/nt database with the default algorithm parameters, taking the LIPI-4 reference sequence as query sequence. The results gave 10 *L. monocytogenes* genome assemblies in additional to one *L. innocua* genome. To introduce more *L. innocua* reference assemblies, all available *L. innocua* assemblies from NCBI Genome database was compared. Two additional *L. monocytogens* genomes which did not contain the LIPI-4 region were also introduced as the reference genome of *L. monocytogenes* lineage -I and lineage -II.

| **Species** | **NCBI Assembly Accession** | **Sample Name** | **Source** |
| --- | --- | --- | --- |
| *L. innocua* | GCF_000241405.1 | ATCC_33091 | *L. innocua* reference |
| *L. innocua* | GCF_000773035.1 | MOD1_LS888 | *L. innocua* reference |
| *L. innocua* | GCF_000960585.1 | 9KSM | *L. innocua* reference |
| *L. innocua* | GCF_000960735.1 | 12KSM | *L. innocua* reference |
| *L. innocua* | GCF_900452995.1 | NCTC12210 | *L. innocua* reference |
| *L. innocua* | GCF_000195795.1 | Clip11262 | BLAST result (complete assembly) |
| *L. monocytogens* | GCF_001565535.2 | LM09-00558 | LIPI-4 reference |
| *L. monocytogens* | GCF_900231485.1 | JF5861 | BLAST result |
| *L. monocytogens* | GCF_000950775.1 | N2306 | BLAST result |
| *L. monocytogens* | GCF_000307085.1 | L312 | BLAST result |
| *L. monocytogens* | GCF_000026705.1 | Clip80459 | BLAST result |
| *L. monocytogens* | GCF_002213965.1 | 02-6680 | BLAST result |
| *L. monocytogens* | GCF_002213505.1 | 02-6679 | BLAST result |
| *L. monocytogens* | GCF_001998985.1 | 10-092876-1063 | BLAST result |
| *L. monocytogens* | GCF_002043045.1 | ICDC-LM188 | BLAST result |
| *L. monocytogens* | GCF_001998945.1 | 10-092876-0168 | BLAST result |
| *L. monocytogens* | GCF_001047715.1 | CFSAN023463 | BLAST result |
| *L. monocytogens* | GCF_001564495.1 | LM05-00008 | *L. monocytogenes* lineage -I reference |
| *L. monocytogens* | GCF_000196035.1 | EGD-e | *L. monocytogenes* lineage -II reference (complete assembly) |
