# Supplementary material for: Whole Genome Analysis of Three Multi-Drug Resistant Listeria innocua and Genomic Insights Into Their Relatedness With Resistant Listeria monocytogenes
Source: Front Microbiol. 2021 Jul 23;12:694361. doi: 10.3389/fmicb.2021.694361 (PMC8343405; doi:10.3389/fmicb.2021.694361)
Supplement: Supplementary file 5 [file Table_3.docx]

**Table S3 Sequence identity and Ka/Ks value**Average nucleotide identity (ANI) among *L. innocua* *(LI)*, *L. monocytogenes* *(LM)* and between two species was calculated using BLAST method. Species-averaged Ka/Ks value for each gene was calculated using γ-MYN algorithm.

| **Gene/ pattern Name** | **Average nucleotide identity %** | | | | **Ka/Ks** | |
| --- | --- | --- | --- | --- | --- | --- |
|  | *LI* | *LM* | Between *species* | p-value | *LI* | *LM* |
| *esaA* | 98.56 | 99.55 | 91.97 | <0.001 | 0.014 | 22.687 |
| *lacE* | 99.68 | 99.90 | 87.23 | <0.001 | 0.041 | 0 |
| *celA* | 99.90 | 100 | 89.30 | <0.001 | 0 | No mutation |
| *lacF* | 99.81 | 100 | 86.73 | <0.001 | 0.118 | No mutation |
| *chbG* | 99.45 | 99.83 | 83.81 | <0.001 | 0.294 | 1.580 |
| *manR* | 99.16 | 99.92 | 83.00 | <0.001 | 0.039 | 1.007 |
| *pagL* | 98.94 | 99.92 | 86.67 | <0.001 | 0.025 | 0.583 |
| *yscE* | 98.46 | 99.25 | 82.41 | <0.001 | 0.080 | 5.002 |
| **LIPI-4** | **99.21** | **99.76** | **85.56** | **<0.001** | **0.061** | **0.516** |
| **Genome** | **98.72** | **99.60** | **89.35** | **<0.001** | **-** | **-** |
